# Life Satisfaction and Instagram Addiction among University Students during the COVID-19 Pandemic: The Bidirectional Mediating Role of Loneliness

**DOI:** 10.3390/ijerph19148414

**Published:** 2022-07-09

**Authors:** Aleksandra M. Rogowska, Patrycja Libera

**Affiliations:** Institute of Psychology, University of Opole, 45-052 Opole, Poland; patrycjalib36@gmail.com

**Keywords:** Instagram addiction, loneliness, mediation analysis, satisfaction with life, social media use, social media addiction, university students, COVID-19 pandemic

## Abstract

Background: Social isolation during the lockdown, and the greater use of online platforms to connect with other people, can alter the dynamic relationship between loneliness, social media use, and subjective well-being. The study examines the mediating role of loneliness in the bidirectional association between Instagram addiction and life satisfaction. Methods: A sample of 954 university students from Poland were enrolled in a cross-sectional online study during the second wave of the COVID-19 pandemic. Participants aged between 19 and 42 (*M* = 22.56, *SD* = 2.36), and most of them were women (86.48%). Standardized questionnaires were used to measure Instagram addiction (BIAS), loneliness (DJGLS), and life satisfaction (SWLS). Results: The prevalence of Instagram addiction, loneliness, and dissatisfaction with life was 17.19%, 75%, and 40.15%, respectively. The mediating effect of loneliness on the relationship between Instagram addiction and life satisfaction was found bidirectionally in women but not among men. Conclusions: Loneliness seems to play a crucial role in the mechanism of social media addiction, so increasing loneliness should be a priority among emerging adults. The target group for intervention and prevention programs at campuses should include lonely and dissatisfied with life university students of the female gender.

## 1. Introduction

Social media has now become an integral part of the daily life of people all over the world. Social network sites (SNS) are used by 90% of adults aged between 18 and 29 [1]. People usually use social media to communicate, exchange information, present themselves, and create their social networks on such websites as Facebook, Instagram, LinkedIn, Nextdoor, Pinterest, Reddit, Snapchat, TikTok, Tinder, Tumblr, Twitter, WhatsApp, or YouTube. A survey conducted in 2021 by the Pew Research Center [2] showed that a majority of U.S. adults use YouTube (81%) and Facebook (69%). However, there are significant differences in the prevalence of SNS use depending on age. For example, Instagram (76%), Snapchat (75%), and TikTok (55%) were the most used SNS among emerging adults 18 to 24 years old [2].

Although the use of the Internet seems to be an inherent attribute of modern man, regardless of geographic location, culture, socioeconomic level, or education, there is a risk of behavioral addiction related to the abuse of this medium. Behavioral addiction can be recognized based on six core components: salience, mood modification, tolerance, withdrawal, conflict, and relapse [3]. The symptoms of Internet addiction can include: (1) excessive use, related to a loss of sense of time or a neglect of basic needs, (2) negative emotions (e.g., tension, anger, depression) when the Internet is inaccessible, (3) increased tolerance, associated with the continuous improvement of Internet and computer parameters, and (4) negative consequences in a social context, including poor performance at school or work, lying and quarrels with loved ones, loss of relationships, social isolation, and fatigue [4]. A compulsive-impulsive spectrum disorder related to Internet use consists of several subtypes, such as social media use, excessive gaming, online shopping, or sexual preoccupations [4,5]. Research shows inconclusive gender involvement in social media addiction [6,7,8,9,10].

The association between the intensity of Instagram use and life satisfaction yielded mixed evidence, as was shown in the systematic review [11]. Some studies reported a small positive association between the intensity of Instagram use and life satisfaction, whereas prospective studies found a negative association between Instagram addiction and life satisfaction [11]. Growing evidence suggests, however, that excessive social media use is associated with low life satisfaction [12,13,14,15,16,17].

Montag et al. [18] suggest that more research is needed to explain the impact of social media use (SMU) on well-being, considering how (occasionally or excessively), who (including age and gender), and why people use social media (e.g., to manage impressions, to share emotions), and also examining an interplay of many important variables. The present study will examine the association between Instagram addiction and life satisfaction among university students regarding gender and a sense of loneliness during the compulsory isolation caused by the coronavirus disease 2019 (COVID-19) pandemic.

Loneliness is a severe prevalent problem associated with adverse somatic and mental health outcomes [19,20,21]. Fox [19] indicated that the prevalence and intensity of loneliness are U-shaped, with a greater value among young adults than in any other age group. However, university students are rarely under scientific consideration.

Loneliness can be considered a severe risk factor for problematic SMU (understood as excessive and passive Internet use), as suggested by O’Day and Heimberg [22]. However, the studies on the relationship between social media use and loneliness are inconsistent [19,23,24,25,26], indicating that many factors may mediate and moderate these associations. Although an ambiguous association is presented between loneliness and non-disordered Internet use, relationships between loneliness with social media addiction (SMA) are consistent across studies. The SMA was found in previous studies as positively related to loneliness [9,10,27,28]. Furthermore, the relationship between SMU and loneliness seems mutual [29,30]. Research suggests that a vicious cycle may start from excessive Internet use, increasing loneliness by withdrawing from face-to-face interactions [31]. In turn, increased loneliness leads to higher Internet use levels and social media addiction by ineffective compensation for poor offline social interactions [28].

Gender can moderate the direct relationship of problematic Internet use (PIU) with loneliness [31,32,33,34]. For example, the positive association between Instagram use and loneliness was found among Turkish male high school students only, while this link was insignificant for females [35]. The other studies also found a marginally stronger association between SMU and loneliness in men than in women [31,32]. In contrast, female gender (β = 0.42), and higher social media use disorder (β = 0.03) were predictors of loneliness among Lebanese adults [36]. Moreover, female gender and single relationship status were related to higher SMU and a greater sense of loneliness among US college students [21]. Among Brazilian university students, smartphone addiction was more prevalent among women and lonely people [33]. However, the studies on gender differences in loneliness–SMU association are still limited [28].

### The Current Study

The current study examines associations between Instagram addiction (IA), loneliness, and life satisfaction among university students from Poland regarding gender differences. Most previous studies on SNS addiction focused on Facebook or general SNS score scores (independent of a specific platform). However, each SNS has unique features, use habits, motives, and gratifications, as suggested by Alhabash and Ma [35]. Therefore, each SNS should be studied separately to explain its specific impact on mental health. The growing prevalence of excessive Instagram use among emerging adults [2,7] requires more research to elucidate the mechanisms and find essential factors influencing these particular SNS.

This study will examine for the first time the bidirectional mediating effect of loneliness on the relationships between SMU and well-being among university students during the general quarantine related to the COVID-19 pandemic. It is unclear whether Instagram use leads to improving or decreasing life satisfaction during pandemic-related social isolation since the results of previous research are mixed. Furthermore, the mechanism of the association between Instagram use and life satisfaction, and the mediating role of loneliness, is not fully explained. Whether loneliness is a predictor of SMA or SMA determines loneliness has been a pending debate in recent years [22]. Researchers postulate that more studies are needed to elucidate potential bidirectional relationships between these associations. Therefore, the indirect relationship between Instagram use and satisfaction with life via loneliness will be tested in this study bidirectionally. The sample of university students was selected to examine the mediation model because this group is at a higher risk of loneliness due to the U-shaped pattern of prevalence [19].

The hypothesis of the mediating role of loneliness on the relationship between subjective well-being and Instagram addiction is based on the cognitive-behavioral model of pathological Internet use (PIU) proposed by Davis [37] and developed further by Caplan [38] as a social skills model of problematic Internet use (PIU). Situational cues (such as the COVID-19 pandemic) and psychopathology predispositions (e.g., depression, social anxiety, substance addiction) affect maladaptive cognitions as distal factors. In contrast, social isolation can directly determine generalized pathological Internet use (GPIU) [37] as a proximal factor of behavioral symptoms of PIU. Both Internet-specific models (ISM) assume that lonely and unhappy people are more likely (than psychosocially healthier individuals) to use and abuse social interaction via social media (including Instagram), which, in turn, leads to adverse outcomes related to Internet use [37,38,39].

According to the displacement hypothesis [28,40], engagement in social media increases loneliness levels because of the displacement of offline relationships and activities with online ones. The Interaction of Person-Affect-Cognition-Execution (I-PACE) model is a theoretical framework for the process underlying the development and maintenance of a specific internet-use disorder [39,41]. Based on the I-PACE model, we can hypothesize that specific Internet-use disorders (such as Instagram addiction) can stabilize and intensify the sense of loneliness (as a dimension of social cognition). The social cognition dimensions interact with a person’s core characteristics that interact with psychopathology, such as depression or social anxiety (as an opposite pole of well-being and life satisfaction). On the other hand, Instagram addiction can directly stabilize and intensify unhappiness and anxiety, leading to worsened life satisfaction. When gratification levels of Instagram use decrease, compensation levels simultaneously increase, leading to behavioral addiction [41]. The displacement hypothesis [28,40] and the I-PACE model [39,41] explain the mediating role of loneliness in the relationship between Instagram addiction and low life satisfaction.

Some previous studies showed that SMU is a positive predictor of loneliness [15,16,31,36,42,43,44], while other research indicated a negative relationship [24,45,46,47] or no significant association [48]. In addition, active and not intensive SMU was a positive predictor of life satisfaction [17,46], while excessive and passive SMU was negatively related to well-being [11,12,13,14,15,16,17]. Loneliness was found as a negative predictor of life satisfaction in previous studies [15,16,47,49], which was also presented during the COVID-19 pandemic [50]. Finally, the mediating effect of loneliness on the relationship between SMA and life satisfaction was found previously in regards to SNS [47], Instagram addiction [16], Facebook addiction [43], and problematic SMU [15], but these studies were not performed during the COVID-19 pandemic-related lockdown when compulsory social isolation was presented in a general population. However, some inconsistency was shown in the associations in particular studies. When addiction to social media or problematic use was examined in the mediation model, SMA increased loneliness, which worsened life satisfaction [15,16,43]. In contrast, if no-disordered SNS was tested, SNS decreased loneliness, increasing life satisfaction [47]. Because the mediation analysis results are inconclusive, it is interesting which one model of mediation will be confirmed (if any) during the COVID-19 pandemic crisis. Research indicates a significant change in worsening mental health was reported among university and college students during the successive waves of the COVID-19 pandemic [51,52,53]. However, it is unclear whether compulsory common isolation also determined the dynamic association between SMU, loneliness, and life satisfaction during the COVID-19 pandemic.

**Figure 1:**
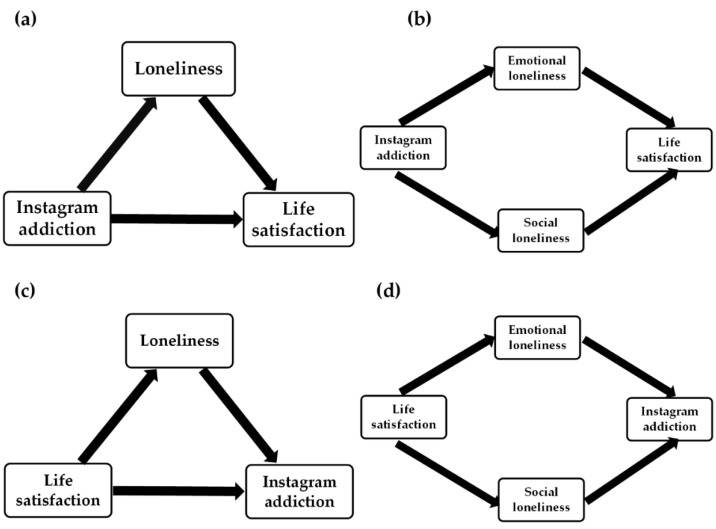
Hypothetical mediating role of loneliness in the relationship between: (**a**) Instagram addiction and life satisfaction (single mediation); (**b**) Instagram addiction and life satisfaction (parallel mediation); (**c**) life satisfaction and Instagram addiction (single mediation); (**d**) life satisfaction and Instagram addiction (parallel mediation).

The indirect effect of life satisfaction on SMUs through loneliness has never been studied to the best of our knowledge. Błachnio et al. [54] found that low life satisfaction can predict high loneliness. Moreover, a high SMU can be predicted by both increased loneliness [21,22,23,30,47,55] and low satisfaction with life [54,55]. Therefore, we expect that loneliness mediates the relationship between life satisfaction and Instagram addiction. Because loneliness is composed of two scales (emotional and social loneliness), two models of mediation will be examined: the simple mediation model with one composite loneliness score (Figure 1a,c) and the parallel mediation model with both emotional and social subscales of loneliness (Figure 1b,d). As a sensitivity analysis, the moderating role of gender was tested for the mediation models. The expected mediating effect of loneliness on the relationship between Instagram addiction and life satisfaction is shown in Figure 1a (single mediation model) and Figure 1b (parallel mediation model). In the opposite direction, the indirect effect of life satisfaction on Instagram addiction via loneliness is presented in Figure 1c (single mediation model) and Figure 1d (parallel mediation model).

## 2. Materials and Methods

### 2.1. Procedure

A cross-sectional survey was conducted online using the Google Forms platform in November and December 2020, with a convenience sample of university students from Poland. The eligibility criterion was: to be a university or college student and be 18 years of age or older, which was examined post-factum in the survey based on the responses in the socio-demographic part of the questionnaire. The first page of the survey contained information about the study and informed consent. The questionnaire could be completed by those participants who agreed to participate in the study and declared that they were students. The participants completed the questionnaire within 10 min on average. The invitation to the survey was posted on the Facebook platforms in student groups at all universities in Poland. A snowball sampling method was also performed because students were asked to post the study invitation to their private Facebook groups. Initially, 956 people responded, but one person refused to participate in the study, and another was 15 years old, so they were removed from further analysis. A total of 954 people were included in the statistical analysis. No missing data was found as the questions were mandatory to answer in the Google Form.

### 2.2. Measurement

#### 2.2.1. Instagram Addiction

Instagram addiction was measured using the six-point modified Bergen Facebook Addiction Scale (BFAS, [56]) in the Polish adaptation and validation [57,58]. In the current Bergen Instagram Addiction Scale (BIAS), the word “Facebook” was replaced with “Instagram”, which was used previously [34]. Each question on the scale addresses one of the six symptoms experienced over the previous 12 months, respectively, to the addiction criteria developed by Griffiths [3]. The answer can be assessed on a 5-point Likert scale (1 = *very rare* to 5 = *very common*). Higher scores can be interpreted as a more positive attitude towards Instagram and a higher risk of Instagram addiction. The cut-off for BFAS ≥ 24 was considered addictive behavior [59]. The reliability of BIAS was assessed using the internal consistency of Cronbach’s α = 0.87, which is even higher than the previously achieved reliability coefficient (Cronbach’s α from 0.82 to 0.86) for BFAS [56].

#### 2.2.2. Loneliness

Loneliness was measured using an 11-items de Jong Gierveld Loneliness Scale (DJGLS, [60]) in the Polish adaptation [61]. Participants rated on a 5-point Likert scale how much they agreed with the sentence (1 = *none of the time*, 2 = *rarely*, 3 = *some of the time*, 4 = *often*, 5 = *all of the time*). The neutral and positive answers are coded as 1 on the emotional loneliness scale (items 2, 3, 5, 6, 9, and 10), characterizing missing relationships. In contrast, the neutral and negative answers are coded as 1 on the social loneliness scale (items 1, 4, 7, 8, and 11), describing belongingness [62]. The total loneliness score is the sum of all dichotomized items, and a higher score indicates a more heightened sense of loneliness in four categories: not lonely (a score of 0–2), moderately lonely (3–8), and strongly lonely (9–11) [63]. In emotional and social subscales, scores equal to higher than 3 means emotional or social loneliness, respectively [63]. The internal consistency of the scale (Cronbach’s α) ranges from 0.80 to 0.90 [62], while in the present sample, Cronbach’s α was 0.91, 0.86, and 0.84 for general, emotional, and social loneliness scales.

#### 2.2.3. Life Satisfaction

The Satisfaction with Life Scale (SWLS, [64]) was developed to measure the global cognitive assessment of satisfaction with various aspects of life in Polish adaptation [65]. Responses to the 5-item SWLS are rated on a 7-point Likert scale (1 = *strongly agree* to 7 = *strongly agree*). The score ranges from 5 to 35 points, and the higher the score, the greater the feeling of satisfaction with life. Total scores ranged from 5 to 35 and can be categorized as extremely dissatisfied (scores between 5 and 9), dissatisfied (10–14), slightly dissatisfied (15–19), neutral (a score of 20), slightly satisfied (21–25), satisfied (26–30), and extremely satisfied (31–35). The reliability coefficient of Cronbach’s α ranged from 79 to 89 in previous studies [66], while in the current study sample, Cronbach’s α was 0.88.

### 2.3. Participants

A sample of 954 university students participated in the study, aged between 19 and 42 (*M* = 22.56, *SD* = 2.36). Among participants, prevailed women (*n* = 825, 86.48%) over men (*n* = 129, 13.52%). Participants represented 87 fields of study and almost 117 various universities in Poland, specializing in technical, economical, humanistic, or art studies. Among students, 32.08% (*n* = 306) studied in the first year of study, 19.08% (*n* = 182) in the second year, 20.02% (*n* = 191) in the third year, 13.31% (*n* = 127) in the fourth year, 15.09% (*n* = 144) in the fifth year, and 0.41% (*n* = 4) in the sixth year. Students represented various levels of study: first level bachelor (*n* = 380, 39.83%), first level bachelor engineering (*n* = 163, 17.09%), second level master (*n* = 190, 19.92%), five-years master (*n* = 216, 22.64%), and third level doctoral (*n* = 5, 0.52%). Full-time study represented 86.90% (*n* = 829) of the total sample, while in the part-time study—13.10% (*n* = 125).

### 2.4. Statistical Analysis

The initial descriptive statistics analysis showed good psychometric properties (skewness and kurtosis ranged between ± 1) for Instagram addiction, life satisfaction, and loneliness (general, emotional, and social). Considering a large sample size (*N* = 954), parametric tests were conducted in the study. The independent samples Student’s *t*-test was used for testing gender differences in Instagram addiction, life satisfaction, and loneliness. The Cohen’s *d* coefficient was used to assess effect size (small for *d* = 0.20, medium when *d* = 0.50, and large if *d* = 0.80).

The association between variables was assessed using Spearman’s correlations because such non-parametric variables were included in the correlation matrix age and gender. The generalized linear modeling (GLM), with a maximum likelihood (ML) estimation method, was used to examine the mediating role of loneliness on the bidirectional association between Instagram addiction and life satisfaction among university students during the COVID-19 pandemic. Bias-corrected percentile bootstrapping method (with 1000 sample replications) was implemented to examine total, direct, and indirect effects in mediation models. As a preliminary analysis, the confirmatory factor analysis (CFA) with the ML estimation method was conducted for Model A mediation (with loneliness as a single measure of general loneliness) and Model B (with emotional loneliness and social loneliness subscales) to check the validity of both models. Models A and B were evaluated using the following goodness-of-fit criteria: ML χ^2^, *df* and *p*-value (the ratio χ^2^/*df* < 5 representing good fit), root mean square error of approximation (adequate fit if RMSEA ≤ 0.08), and comparative fit index (CFI ≥ 0.90 meaning adequate fit) [67]. Moderated mediation was performed to examine gender roles in the mediation models, using Model 7 of PROCESS ver. 3.5. Macro for SPSS, designed by Hayes [68,69]. The conditional effect was examined based on a percentile bias-corrected bootstrapping procedure with 1000 samples. A bootstrap confidence interval (95% *CI*) not including “0” signals a significant effect.

The CFA analysis was conducted using AMOS ver. 26 for the IBM SPSS software ver. 26 [70]. The GLM analysis and visualization of the correlation matrix for men and women (Figure S1 in Supplementary Materials) were assessed using JAMOVI software ver. 25 [71].

## 3. Results

### 3.1. Prevalence of Instagram Addiction, Life Satisfaction, and Loneliness among University Students during the COVID-19 Pandemic

In the students’ sample, prevalence of excessive Instagram addiction (BIAS ≥ 24) was 17.19% (*n* = 164). Regards life satisfaction, there was 6.08% (*n* = 58) extremely dissatisfied individuals, 14.68% (*n* = 140) dissatisfied, 19.39% (*n* = 185) slightly dissatisfied, 5.03% (*n* = 48) of neutral respondents, 29.04% (*n* = 277) slightly satisfied, 20.13% (*n* = 192) satisfied, and 5.66% (*n* = 54) of extremely satisfied. Overall, 40.15% (*n* = 383) people were dissatisfied with their life, from slight to extremely strong levels (SWLS ≤ 19). Among university students, 24.84% (*n* = 237) was not lonely, while 48.53% (*n* = 463) was moderately lonely, and 26.63% (*n* = 254) was strongly lonely. Emotional loneliness reported 61.22% (*n* = 584) of participants, whereas social loneliness 46.54% (*n* = 444).

### 3.2. Descriptive Statistics for Instagram Addiction, Loneliness, and Life Satisfaction

Initially, descriptive statistics were performed for Instagram addiction, life satisfaction, and loneliness (general loneliness and two subscales: emotional and social loneliness) to check assumptions for parametric tests (Table S1 in the Supplementary Materials). Since skewness and kurtosis ranged between ±2 and the large sample (*N* = 954), parametric statistics were conducted in the following steps. The independent samples Student’s *t*-test examined whether gender differences exist in such variables as Instagram addiction, life satisfaction, and loneliness (Table 1). Significant differences were only found for Instagram addiction, with a medium effect size (Cohen’s *d* = 0.62). Women scored higher than men in excessive Instagram use. No gender differences were presented in loneliness scales (general, emotional, social loneliness) and satisfaction with life (Table 1).

A Spearman’s correlations were performed to examine the association between demographic variables such as gender and age, Instagram addiction, life satisfaction, and loneliness (Table 2). Instagram addiction is related to the female gender, negatively associated with life satisfaction, while positively linked with all scales of loneliness: general, emotional, and social. All loneliness scales are negatively related to life satisfaction while positively to Instagram addiction. Younger age is related to higher scores in general and emotional loneliness. Life satisfaction is negatively associated with Instagram addiction and all three scales of loneliness (general, emotional, and social), while is unrelated to gender and age. All correlations showed a weak or medium strength. Loneliness subscales were strongly associated with each other.

For sensitivity analysis, the correlational matrix was performed separately for women and men (Figure S1 in Supplementary Material). The analysis showed that Instagram addiction is significantly related to all three scales of loneliness (general, emotional, and social) among women, but is unrelated to any one of the loneliness scales in men. Although life satisfaction was related to loneliness (all three scales) in both gender groups, the correlation was strengthened in women than in men (Figure S1).

### 3.3. The Preliminary Analysis of the Validity of the Measurement Models

Two different models will be examined using structural equation modelling (SEM): (1) Model A with Instagram addiction (6 items), life satisfaction (5 items) and loneliness general (11 items); and (2) Model B with Instagram addiction (6 items), life satisfaction (5 items), and two subscales of loneliness: emotional loneliness (6 items), and social loneliness (5 items). The Model A with general loneliness scale showed very good fit, including χ^2^ = 421.264, *df* = 179, χ^2^/*df* = 2.353, RMSEA = 0.038, SRMR = 0.045, CFI = 0.973. Moreover, validity was very good, indicating that all variable measures are consistent internally (Cronbach’ α > 0.07), have good convergent validity (CR > 0.70, AVE > 0.50, *r* < 0.80) and each construct differ each other (considering MSV < AVE, Fornell–Larcker criterion: the square root of the AVE > *r*, and HTMT > 0.70) showing distinct characteristics of participants (Table S2 in the Supplementary Materials).

The Model B, with two separate scales emotional loneliness and social loneliness, demonstrated yet better fit than the first model: χ^2^ = 412.340, *df* = 177, χ^2^/*df* = 2.330, RMSEA = 0.037, SRMR = 0.046, CFI = 0.974. However, both scales of loneliness did not show a sufficient convergent validity, considering AVE was less than 0.50, and the correlation between emotional and social loneliness was greater than 0.80 (Table S3 in the Supplementary Materials). In addition, the discriminant validity was not appropriate for both loneliness scales since AVE was less than MSV, and the square root of the AVE for emotional loneliness was less than the correlation with social loneliness, showing that emotional loneliness is not sufficiently distinct from social loneliness. Therefore, parallel mediation analysis, with both scales of emotional loneliness and social loneliness, was not performed in the further stage of statistical analysis.

### 3.4. The Bidirectional Mediating Effect of Loneliness on the Instagram Addiction—Life Satisfaction Association

The bidirectional relationship between life satisfaction and Instagram addiction was examined in two separate mediation models (Model 1 and Model 2), using the GLM mediation analysis. Model 1 examines the indirect effect of Instagram addiction on satisfaction with life via loneliness (Figure 1a). Results are presented in Table 3. People who used Instagram excessively presented higher loneliness scores, which in turn decreased the life satisfaction of university students during the COVID-19 pandemic. Simple mediation showed that all effects (total, direct, and indirect) are significant, which means that loneliness partially mediates the association of excessive Instagram addiction with life satisfaction. Model 1 explains 19% of life satisfaction variance (*R*^2^ = 0.19).

The opposite direction of the relationship between life satisfaction and Instagram addiction via loneliness was tested as Model 3 of mediation (Figure 1c). A low level of life satisfaction leads to high loneliness, which in turn increases the risk of Instagram addiction among university students during the COVID-19 pandemic (Table 4). Since total, direct, and indirect effects are significant, we can conclude that loneliness partially mediates the association between life satisfaction and Instagram addiction. However, Model 2 of regression explains only 7% of Instagram addiction variance.

### 3.5. The Moderating Role of Gender in Model 1 of Mediation

Moderated mediation of Model 1 was performed in the study to examine whether women and men differ in mediating the effect of loneliness on the Instagram addiction–life satisfaction association. As shown in Table 5, the mediating effect of loneliness on the relationship between Instagram addiction and life satisfaction is demonstrated only in women but not among men. All connections between Instagram addiction, loneliness and life satisfaction, and loneliness and life satisfaction were significant among women (Table 5). However, in men, a low life satisfaction level can be predicted only by a high sense of loneliness, but not by Instagram addiction.

**Figure 2:**
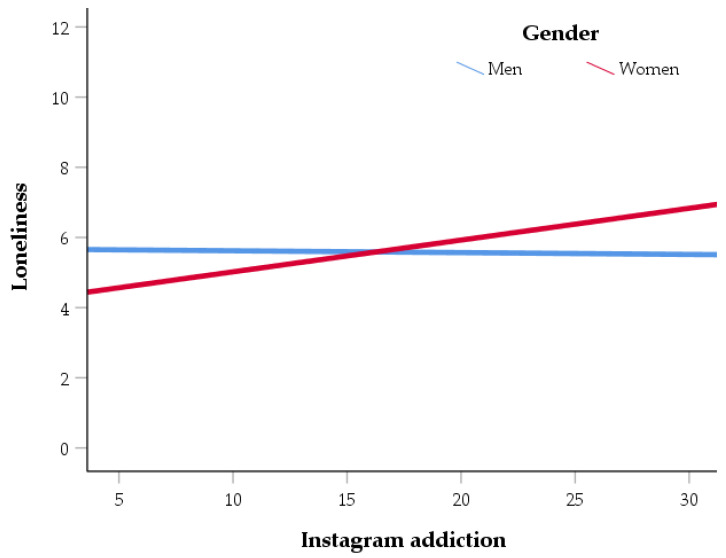
Interaction effect between Instagram addiction and gender on loneliness.

Although gender solely has no significant effect on loneliness (*b* = 0.16, *SE* = 0.37, *t* = 0.04, *p* = 0.97, Boot *M* = 0.02, *SE* = 0.35, *CI* = −0.661; 0.705), interaction effect between gender and Instagram addiction on loneliness was significant, considering bootstrapping (Boot *M* = 0.10, *SE* = 0.05, *CI* = 0.000; 0.193), but insignificant when regression was examined (*b* = 0.10, *SE* = 0.05, *t* = 1.78, *p* = 0.08). Further bootstrap analysis showed that the conditional indirect effect of Instagram addiction on life satisfaction via loneliness was significant for women (Boot effect = −0.07, *SE* = 0.02, *CI* = −0.107; −0.042), but insignificant in men (Boot effect = 0.00, *SE* = 0.04, *CI* = −0.068; 0.076). Model 1 moderated mediation can explain 19% of Instagram addiction variance, *R* = 44, *R*^2^ = 0.19, *F*(2, 951) = 110.75, *p* < 0.001. Figure 2 shows the moderating effect of gender on the association between Instagram addiction (predictor) and loneliness (dependent variable and component of mediation analysis). Higher Instagram use can be predicted by higher loneliness in women, while no relationship was found in men.

### 3.6. The Moderating Role of Gender in Model 2 of Mediation

Analysis of moderated mediation was conducted again to examine the conditional effect of life satisfaction on loneliness regarding gender. The results indicate that mediating effect of loneliness on the relationship between life satisfaction and Instagram addiction is presented in women but does not exist in men (Table 6). The only significant negative association was found between life satisfaction and loneliness among men, suggesting that a low level of life satisfaction can predict their high loneliness. In contrast, associations between all three variables (life satisfaction, loneliness, and Instagram addiction) were significant in women (Table 6).

**Figure 3:**
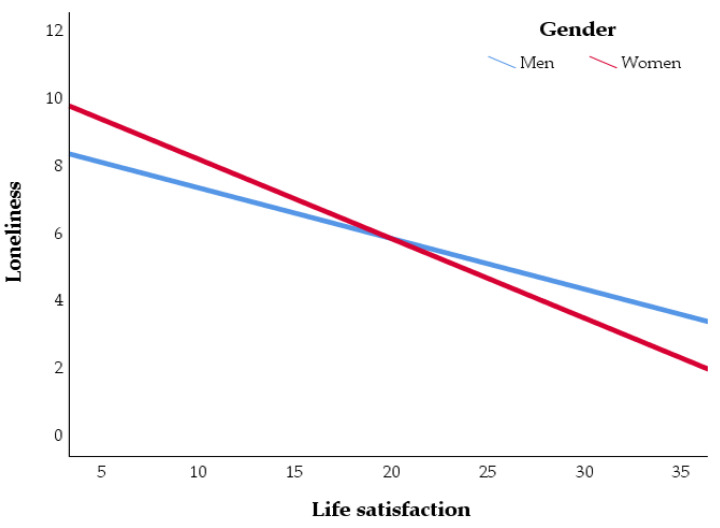
Interaction effect between life satisfaction and gender on loneliness.

The study did not find neither gender association with loneliness (*b* = −0.07, *SE* = 0.30, *t* = −0.22, *p* = 0.83, Boot *M* = −0.07, *SE* = 0.31, *CI* = −0.674; 0.529). The interaction effect was presented between life satisfaction and gender on loneliness, considering regression results (*b* = −0.09, *SE* = 0.04, *t* = −1.98, *p* = 0.48), but bootstrapping did not confirm it (Boot *M* = −0.82, *SE* = 0.05, *CI* = −0.182; 0.009). Although a negative relationship between life satisfaction and loneliness seems stronger in women than in men, this tendency may be not conclusive (see Figure 3 for more details). Model 2 moderated mediation can explain only 3% of Instagram addiction variance, *R* = 16, *R*^2^ = 0.03, *F*(2, 951) = 12.62, *p* < 0.001. 

## 4. Discussion

The present study examined the mediating role of loneliness in bidirectional life satisfaction–Instagram addiction association during the COVID-19 pandemic for the first time. The research was performed when university students were isolated from teachers and friends participating in remote online education. The lockdown was related to several restrictions, including social isolation, wearing masks on the face and one-time gloves, and avoiding people in shops and social centers to prevent a coronavirus contagion. These restrictions and uncertain futures related to academic achievement, economic status, or work and housing maintenance contributed to the worsening well-being of university students [50,51,52,53].

It could be assumed that the social isolation situation could increase loneliness [72], and SMU will be used as a medium to compensate lack of offline social interactions and loneliness during the COVID-19 pandemic-related lockdown. Indeed, the research indicates that during the COVID-19 pandemic, younger adults (aged 18–34) were more lonely than older participants, and higher loneliness was predicted in those who reported more increased SMU [42]. Loneliness also decreased life satisfaction among young adults from South Africa during the COVID-19 pandemic [50]. Unfortunately, the excessive use of social media may contribute to developing an addiction by starting a vicious circle through loneliness-SMU reciprocal links used by people to heighten well-being. This study explored the links between Instagram addiction, loneliness, and life satisfaction among university students to find the mechanism responsible for the development of addictive behaviors and to prevent in the future such adverse consequences of the pandemic.

### 4.1. Prevalence of Internet Addiction, Loneliness, and Life Satisfaction among University Students during the COVID-19 Pandemic

The prevalence of severe Instagram addiction was 17.19% in this study, using a cut-off score ≥ 24 on the Bergen Instagram Addiction Scale (BIAS). The Bergen Social Media Addiction Scale (BSMAS) is one of the most frequently used questionnaires to assess social media addiction, consisting of six items concerning six criteria of compulsive behavior, such as preoccupation, tolerance, withdrawal, persistence, escape, and conflict [73]. A meta-analysis on the prevalence of SMA (assessed using this questionnaire across 32 countries) found 23% among homogenous samples of university students [59]. The pooled prevalence of social media addiction was 8% in ten studies when a cut-off score ≥ 24 was used [59]. However, findings indicate that the prevalence rate is dependent on several factors, including the method of cut-off score classification (e.g., monothetic or polythetic), country (higher prevalence in collectivist nations than individualist nations), sample size, and age (higher prevalence in the younger generation). Although the prevalence was referred to the same questionnaire, usually Facebook addiction (BFAS) or general social media addiction (BSMAS) was reported in the meta-analysis [59], which may not be appropriate to Instagram addiction.

Previous research indicated that the preference for social media use might vary between particular generations and countries. Various expectations for SNS usage were found depending on the generational cohort (i.e., baby boomers, generation X, and millennials), which can explain generational differences in specific SNS preferences [74]. Among various SN, Instagram and Facebook were the most frequent among college students in Spain [9]. A recent international study showed that 8% of adolescents (aged 11–16) from 44 countries reported symptoms of social media addiction, with high disparities in prevalence rates among countries [14]. Recently, a particular increase in interest among young people has been noted in image-sharing SNS, such as Instagram [75]. The primary motivation for sharing photos is the need for self-representation and status-seeking [76]. Among American Instagram users, most reported visiting the platform daily, including 38% several times a day, 21% about once a day, and 41% less frequently [2]. Among emerging adults 18–24-year-olds, 76% reported using Instagram [2]. Furthermore. Instagram was used predominantly among Hispanics (52%) and Black Americans (49%), compared with White Americans (35%). More research is necessary on the prevalence of Instagram addiction to interpret the present results.

Loneliness is a severe prevalent problem in today’s networked society and is associated with various somatic and mental health problems [19]. A systematic review showed that loneliness had been a significant issue during the COVID-19 pandemic, which contributed to mental health problems and a decrease in the well-being of populations worldwide [77]. Most studies reported increased loneliness during the COVID-19 pandemic compared to the pre-pandemic period, as shown in a systematic review [78]. In the present sample, 48.53% were moderately lonely. In comparison, 26.63% were strongly lonely, using a cut-off score > 3 in the DJGLS, indicating a prevalence of 75% of moderate to severe loneliness among Polish university students during the COVID-19 pandemic. The results are consistent to some degree with a previous study among the general population from Germany, in which 30% were not lonely, 44% were rarely lonely, and 26% reported some degree of loneliness [79]. Moreover, a multi-country study indicates that severe loneliness was reported in 21% of adults from 101 countries during the COVID-19 pandemic, which is close to the present study [80]. In contrast, a much lower prevalence of medium-high levels of loneliness (45%) was found previously among college students from Spain [9]. Significant heterogeneity in the prevalence of loneliness was found previously in a systematic review [78], which seems to explain some disparities in the prevalence between the present and previous studies.

Among university students in this study, 40.15% were dissatisfied with their life slightly to an extreme level. Similar results (42.33%) were found in a previous study performed among university students from Poland during the COVID-19 pandemic [51]. However, the prevalence rates changed significantly between the next pandemic waves (W), indicating 37.44%, 40.06%, and 49.54% of unsatisfied individuals in the W1, W2, and W3, respectively [51]. The present study is in line with the international research, which showed the prevalence rate of 39.46% unsatisfied with life university students during the first wave of the COVID-19 pandemic, with vast differences, however, between nine countries [52]. More research is needed to compare the present prevalence rates of university students from Poland with those from other regions of the world.

### 4.2. Bidirectional Associations between Instagram Addiction, Loneliness, and Life Satisfaction

Instagram addiction was positively correlated to loneliness while negatively to life satisfaction among university students during the COVID-19 pandemic. In addition, loneliness correlated negatively with life satisfaction in this study. Furthermore, the bidirectional relationship between life satisfaction and Instagram addiction was confirmed in this study. Consistent with previous studies, this study found that low life satisfaction can be predicted by high Instagram addiction [11,12,13,14,15,16,17] and high loneliness [15,16,43,47,49]. A high Instagram addiction is a predictor of high loneliness in this study, which is also in line with most previous studies [15,16,31,36,42,43,44]. Finally, the negative direct and indirect relationship between Instagram addiction and life satisfaction via loneliness was found in this study. Model 1 of mediation is consistent with previous findings [15,16,43].

There is a large body of literature that support the present results. A negative association was also found between life satisfaction and problematic social media use among adolescents from 40 countries, with an effect size from small to large, depending on the country [14]. Excessive SMU decreased subjective well-being among a nationally representative sample of Finnish social media users [15]. Instagram addiction was related to low life satisfaction among Malaysian undergraduates [16]. Furthermore, Błachnio et al. [12] showed that life satisfaction depends on the intensity of Facebook use (excessive use and Facebook addiction were negatively related to life satisfaction). Excessive SMU and time spent browsing the web are positively associated with loneliness and negatively related to life satisfaction, as indicated by cross-sectional findings [44]. Among high school students from Portugal, those who spend free time on social networks and simultaneously enjoy being alone were not satisfied with their life [32]. Instagram addiction was directly negatively related to life satisfaction and positively to loneliness. In contrast, loneliness was a negative predictor of life satisfaction, confirming a mediating role of loneliness in the Instagram addiction–life satisfaction association [16]. An indirect association between PSMU and life satisfaction via loneliness was also confirmed previously in a longitudinal study [15]. A longitudinal study among nationally representative Finnish social media users showed that loneliness plays a crucial mediating role in the relationship between problematic social media use (PSMU) and life satisfaction [15]. Increasing in PSMU did not predict decreased satisfaction with life. However, increased PSMU predicted increased loneliness, which in turn predicted reduced satisfaction with life. Satici [43] also showed that loneliness fully mediates the relationship between Facebook addiction and subjective well-being. This study confirms for the fourth time the crucial role of loneliness in the association between SMA and satisfaction with life.

For the first time, however, to our best knowledge, the opposite direction of mediation was found in this study. The mediating effect of loneliness on the relationship between life satisfaction and Instagram addiction was also examined for the first time during the COVID-19 pandemic. Consistent with previous studies, Instagram addiction can be predicted by low life satisfaction [54,55], as well as high loneliness [21,22,23,30,31,55]. Moreover, high loneliness can be predicted by a low life satisfaction level, which was also found in another study [54]. Finally, a partial mediating effect of loneliness on the relationship between life satisfaction and Instagram addiction was presented in this study. Model 2 mediation was expected, based on previous findings, but it was never yet examined.

Previous research showed similar bivariate associations between variables in the mediation Model 2, suggesting that mediation analysis is appropriate. Among Polish users of mobile phones [54], the structural equation modeling (SEM) showed that a low level of satisfaction with life increases loneliness and Facebook intrusion. Furthermore, low loneliness predicted high Facebook intrusion, increasing loneliness levels. In the other studies, Błachnio et al. [23] found that loneliness was a positive predictor of Facebook usage in a sample of young Polish adults. Loneliness positively and life satisfaction negatively predicted problematic internet use among UK adult participants working from home during the UK COVID-19 lockdown [55].

This is important to note that Model 1 explains only 19% of life satisfaction variance. Although the direct and indirect effects were significant, Model 2 explains only 7% of Instagram addiction variance, and this value decreased to 3% when gender was included in the moderated mediation model. Therefore, some other important variables (not included in Model 1 and Model 2) can contribute to Instagram addiction to a greater extent. Several factors may affect the association between SMU and well-being. More research is necessary to explain fully the association between Instagram addiction and life satisfaction among university students.

The present study showed that low life satisfaction and excessive Instagram use are bidirectional risk factors for one another. However, it is important to note that sensitivity analysis of moderated mediation showed that mediation models were presented exclusively in women but not men, which will be discussed in the next section. Moreover, because excessive Instagram use seems to more strongly predict poor well-being (β = −0.14. total effect) than vice versa (β = −0.13. total effect), excessive Instagram use may be prodromes for low levels of life satisfaction. An explanation of the bidirectional and dynamic relationship between loneliness and SMU may base on the pivotal role of motivation to use social media [28]. Loneliness increases when SMU is seen as a way to escape the offline social world. In contrast, when SMU is a way to expand one’s social connections and strengthen existing ones, loneliness levels would decrease.

Model 2 in this study also seems to be consistent with the cognitive-behavioral model of PIU [37] and a social skills model of PIU [38]. It seems possible that the COVID-19 pandemic favored increasing social media use to communicate with others during social isolation caused by lockdown or during the strict quarantine. Increased Instagram use could be used as a coping strategy to mitigate a sense of loneliness. A higher level of anxiety and depression and a lower level of subjective well-being could support the development of Instagram addiction in vulnerable individuals. Due to both Internet-specific models [39], it was expected that high loneliness and low life satisfaction would be linked to high scores in Instagram addiction [38,39].

On the other hand, increased use of social media raises loneliness, according to the displacement hypothesis [28,40]. Model 1 of mediation was confirmed, at least to some extent, with the I-PACE model developed by Brand et al. [41]. The process of specific Internet-use disorders is the consequence of interactions between predisposing factors (e.g., neurobiological and psychological constitutions, including the social perception of loneliness), moderators (e.g., coping styles and Internet-related cognitive biases, such as expectancies, illusions, or implicit associations), and mediators (e.g., affective and cognitive responses to situational triggers in combination with reduced executive functioning). An addiction process is based on increased strength of conditioning via mechanisms of the shift from gratification (high at early stages and decreased during the addiction process) to compensation (low at the earlier process and then successively increased) and contribution of affective and cognitive responses to external and internal triggers. Further study should be conducted longitudinally, including the motivational aspect of SMU to the mediation model.

### 4.3. The Moderating Role of Gender on Mediation Models

This study found that women scored higher than men in Instagram addiction, and the effect size was medium. The correlation between Instagram addiction and female gender was weak but significant (*r*_S_ = 0.22, *p* < 0.001). Furthermore, this study demonstrated clearly, that the association of Instagram addiction with life satisfaction and loneliness is significant exclusively in women, but these variables are unrelated among men. Hence, the mediating effect of loneliness on the reciprocal relationship between life satisfaction and Instagram addiction is also presented in women but does not exist in men. Therefore, the mediation pattern found in this study in Model 1 and Model 2 is moderated by gender.

Although the study on gender roles in SMA is inconclusive [6,7,8,9,10], some studies seem to confirm the present results [6,7]. For example, in the sample of US college students, Instagram was most frequently used among women [7]. Furthermore, female gender and single relationship status were the most important risk factors for excessive SMU among US university students [21]. Moreover, women spend more time on social media than male Jordanian medical students [6]. Similarly, in the sample of Brazilian university students, women and excessive Instagram users had significantly higher smartphone addiction scores [33] than men and users of other social media applications. Andreassen et al. [73] found that men use video games additively. At the same time, women are more likely to use social media excessively, and the single status of the relationship is a risk factor for both genders. Yurdagül et al. [34] suggested that problematic Instagram use (PIU) has different psychopathological outcomes (including loneliness) on male and female adolescents, so gender and age are essential moderators of these associations.

On the other hand, the bidirectional association between loneliness and life satisfaction was presented consistently in the study, independent of gender. The present result is consistent with the longitudinal study performed on the U.S. population for four years [49], which indicated that loneliness could longitudinally predict subjective well-being. Moreover, interventions on lowering loneliness have a one-year effect on subjective well-being, while increases in subjective well-being have a two-year effect on decreasing loneliness. Although asymmetrical feedback was found in this study between loneliness and subjective well-being, an intervention aimed at both variables has substantial psychological and health benefits [49].

There are several possible moderators that can be examined in further studies on the bidirectional relationship between subjective well-being and Instagram use via loneliness. Among sociodemographic variables, possible moderators can include socioeconomic status, relationship status (single, in a relationship), age, generation belonging, country of origin, and ethnicity. Among psychological variables, individual differences in self-esteem, self-attractiveness, self-efficacy, personality traits (such as extroversion, neuroticism, agreeableness, openness), the severity of mental health problems and disorders (such as stress, insomnia, anxiety, depression, PTSD), and dominant coping with stress strategies and styles, can be considered potential moderators. In addition, some characteristics of SNSs may be moderators, including time spent on SNSs, the number, and frequency of various SNSs use (besides Instagram), addictive vs. not-problematic Instagram, and other SNSs use.

### 4.4. Limitation of the Study

The sample size was quite large, and students represent various universities and faculties, suggesting that the study is representative of the university student population in Poland. Although findings are homogenous internally and consistent with previous studies, some limitations do not allow for their generalization. The analysis was performed online, using social media platforms and applications, because of restrictions related to lockdown during the COVID-19 pandemic. However, the results may not be appropriate for people not using social media. Moreover, using social media to invite participants to the study and snowball sampling may be a source of bias. The study was conducted in Poland, so the results may not be generalized to university students from other countries. Cross-national research is necessary for the future to verify the present results in different cultural contexts. Unfortunately, the sample predominated women, so future research should be more balanced regards gender. The self-report measures can also be some limitation in the study as a subjective assessment of SMA. Future studies could objectively measure the time of SMU and types of social media activity to avoid delusional self-beliefs. Finally, the cross-sectional study design does not allow cause-and-effect inference. Therefore, international and longitudinal research on a more representative sample of university students would be advisable. The study examined Instagram addiction regarding selected variables, such as life satisfaction, loneliness, gender, and age. Future studies could consider more factors and potential confounding variables to see Instagram addiction from a more complex perspective. The study did not compare the present results with pre-pandemic time, so it is unclear whether the results are specific to the COVID-19 pandemic or show universal patterns independent of the pandemic crisis. The limitation of this study is also a way of presenting the results, which may not be easy to understand for the reader not familiar with quantitative methods and statistical analyses, as well as for the generalist audience.

## 5. Conclusions

Most university students felt lonely during the COVID-19 pandemic, but simultaneously, most were satisfied with their life and did not show a risk of Instagram addiction. Furthermore, the mediating effect of loneliness was presented bidirectionally on the Instagram addiction–life satisfaction association, but exclusively in women. Loneliness may play a crucial role in the mechanism of social media addiction, but the moderating role of gender must be considered in further studies. Therefore, the intervention and prevention programs at campuses should focus on decreasing loneliness levels among female university students during such global crises as a pandemic.

The bidirectional mediation (Model 1 and Model 2) was presented differently across gender groups. The regression analysis showed that the bidirectional indirect effect of Internet addiction on life satisfaction and life satisfaction on Instagram addiction was presented only in women. The target groups for intervention and prevention programs are women, lonely people, those at high risk of Instagram addiction, and dissatisfied with their life. For men, low or moderate Instagram use can be an excellent strategy to maintain well-being and avoid loneliness by replacing the offline social relationship with active online communication with other people. More research is needed, particularly longitudinal, to verify the present cross-sectional findings.

## Figures and Tables

**Table 1:** Gender differences in Instagram addiction, loneliness, and life satisfaction (*N* = 954).

Variable	Women (*n* = 825)		Men (*n* = 129)		*t*(952)	*p*	*d*
	*M*	*SD*	*M*	*SD*			
Instagram addiction	16.96	6.30	13.07	6.15	6.54	<0.001	0.62
Life satisfaction	20.61	6.70	21.30	7.04	1.08	0.281	0.10
Loneliness general	5.65	3.54	5.61	3.42	0.14	0.890	0.01
Emotional loneliness	3.27	2.10	3.15	2.04	0.63	0.527	0.06
Social loneliness	2.38	1.78	2.46	1.76	0.47	0.638	0.05

**Table 2:** Spearman’s correlations (*N* = 954).

Variable	Age	Gender	Instagram Addiction	Life Satisfaction	Loneliness	Emotional Loneliness
Gender	−0.03					
Instagram addiction	−0.05	0.22 ***				
Life satisfaction	0.04	−0.03	−0.14 ***			
Loneliness	−0.01 **	0.01	0.13 ***	−0.44 ***		
Emotional loneliness	−0.13 ***	0.02	0.16 ***	−0.42 ***	0.93 ***	
Social loneliness	−0.04	−0.02	0.07 *	−0.37 ***	0.90 ***	0.66 ***

Note. Gender was coded: women = 1, men = 0. * *p* < 0.05, ** *p* < 0.01, *** *p* < 0.001.

**Table 3:** Path Model 1 for the simple mediating effect of loneliness on the relationship between Instagram addiction and life satisfaction among university students (*N* = 954).

Type	Effect	*B*	*SE*	95% BCa CI		β	*z*	
				LL	UL			
Indirect	Instagram ⇒ Lonel. ⇒ Life sat.	−0.06	0.02	−0.09	−0.03	−0.06	−4.02	<0.001
Component	Instagram ⇒ Lonel.	0.08	0.02	0.04	0.11	0.14	4.19	<0.001
	Lonel. ⇒ Life sat.	−0.80	0.05	−0.90	−0.69	−0.42	−14.83	<0.001
Direct	Instagram ⇒ Life sat.	−0.07	0.03	−0.13	−0.01	−0.08	−2.30	0.021
Total	Instagram ⇒ Life sat.	−0.14	0.03	−0.21	−0.08	−0.13	−4.19	<0.001

Note. Instagram = Instagram addiction, Lonel. = Loneliness, Life sat. = life satisfaction, CI = confidence interval, LL = lower level, UL = upper level. Confidence intervals computed with Bias corrected bootstrap method (BCa). Betas are completely standardized effect sizes.

**Table 4:** Path model for the simple mediating effect of loneliness on the relationship between life satisfaction and Instagram addiction among university students (*N* = 954).

Type	Effect	*B*	*SE*	95% BCa CI		β	*z*	*p*
				LL	UL			
Indirect	Life sat. ⇒ Lonel. ⇒ Instagram	−0.04	0.01	−0.07	−0.01	−0.04	−2.75	0.006
Component	Life sat. ⇒ Lonel.	−0.22	0.02	−0.25	−0.19	−0.43	−14.50	<0.001
	Lonel. ⇒ Instagram	0.18	0.06	0.06	0.31	0.10	2.85	0.004
Direct	Life sat. ⇒ Instagram	−0.09	0.04	−0.16	−0.02	−0.09	−2.49	0.013
Total	Life sat. ⇒ Instagram	−0.13	0.03	−0.19	−0.07	−0.13	−4.19	<0.001

Note. Instagram = Instagram addiction, Lonel. = Loneliness, Life sat. = life satisfaction, CI = confidence interval, LL = lower level, UL = upper level. Confidence intervals computed with Bias corrected bootstrap method (BCa). Betas are completely standardized effect sizes.

**Table 5:** The moderating role of gender on the indirect effect of Instagram addiction on life satisfaction via loneliness.

Moderator Gender	Type	Effect	*B*	*SE*	95% BCa CI		β	*z*	*p*
					LL	UL			
Men	Indirect	Instagram ⇒ Lonel. ⇒ Life sat.	0.00	0.03	−0.06	0.07	0.00	0.10	0.917
Men	Component	Instagram ⇒ Lonel.	−0.01	0.05	−0.10	0.09	−0.01	−0.11	0.913
Men		Lonel. ⇒ Life sat.	−0.64	0.18	−1.01	−0.31	−0.34	−3.53	<0.001
Men	Direct	Instagram ⇒ Life sat.	0.04	0.11	−0.18	0.25	0.04	0.38	0.707
Men	Total	Instagram ⇒ Life sat.	0.04	0.10	−0.14	0.23	0.04	0.46	0.642
Women	Indirect	Instagram ⇒ Lonel. ⇒ Life sat.	−0.07	0.02	−0.11	−0.04	−0.07	−4.47	<0.001
Women	Component	Instagram ⇒ Lonel.	0.09	0.02	0.06	0.13	0.16	4.75	<0.001
Women		Lonel. ⇒ Life sat.	−0.82	0.06	−0.93	−0.70	−0.43	−13.97	<0.001
Women	Direct	Instagram ⇒ Life sat.	−0.09	0.04	−0.17	−0.02	−0.09	−2.57	0.010
Women	Total	Instagram ⇒ Life sat.	−0.17	0.04	−0.24	−0.09	−0.16	−4.53	<0.001

Note. Instagram = Instagram addiction, Lonel. = Loneliness, Life sat. = life satisfaction, CI = confidence interval, LL = lower level, UL = upper level. Confidence intervals computed with Bias corrected bootstrap method (BCa). Betas are completely standardized effect sizes.

**Table 6:** The moderating role of gender on the indirect effect of life satisfaction on Instagram addiction via loneliness.

Moderator Gender	Type	Effect	*B*	*SE*	95% BCa CI		β	*z*	*p*
					LL	UL			
Men	Indirect	Life sat. ⇒ Lonel. ⇒ Instagram	0.00	0.03	−0.05	0.05	0.00	−0.03	0.976
Men	Component	Life sat. ⇒ Lonel.	−0.15	0.04	−0.23	−0.06	−0.29	−3.38	<0.001
Men		Lonel. ⇒ Instagram	0.01	0.16	−0.31	0.31	0.00	0.03	0.974
Men	Direct	Life sat. ⇒ Instagram	0.03	0.09	−0.16	0.21	0.04	0.37	0.714
Men	Total	Life sat. ⇒ Instagram	0.03	0.08	−0.12	0.19	0.04	0.44	0.663
Women	Indirect	Life sat. ⇒ Lonel. ⇒ Instagram	−0.05	0.02	−0.08	−0.02	−0.05	−2.81	0.005
Women	Component	Life sat. ⇒ Lonel.	−0.24	0.02	−0.27	−0.20	−0.45	−13.94	<0.001
Women		Lonel. ⇒ Instagram	0.20	0.07	0.07	0.34	0.11	2.91	0.004
Women	Direct	Life sat. ⇒ Instagram	−0.10	0.04	−0.18	−0.02	−0.11	−2.52	0.012
Women	Total	Life sat. ⇒ Instagram	−0.15	0.03	−0.21	−0.08	−0.16	−4.58	<0.001

Note. Instagram = Instagram addiction, Lonel. = Loneliness, Life sat. = life satisfaction, CI = confidence interval, LL = lower level, UL = upper level. Confidence intervals computed with Bias corrected bootstrap method (BCa). Betas are completely standardized effect sizes.

## Data Availability

Data supporting reported results can be found at Mendeley Data: Rogowska, A. The relationship between Instagram addiction, loneliness, and life satisfaction among university students from Poland, during the second wave of the COVID-19 pandemic. Mendeley Data, 2022, V1, doi:10.17632/672kbpfdh3.1 (available online at https://data.mendeley.com/drafts/672kbpfdh3; accessed on 15 June 2022).

## Supplementary Materials

The following supporting information can be downloaded at: https://www.mdpi.com/article/10.3390/ijerph19148414/s1, Table S1: Descriptive statistics (*N* = 954); Table S2: Model A validity measures for Instagram addiction, life satisfaction, and general loneliness (*N* = 954); Table S3: Model B validity measures for Instagram addiction, life satisfaction, emotional loneliness, and social loneliness (*N* = 954); Figure S1: Correlation matrix for gender differences (Men = 0, *n* = 129; Women = 1, *n* = 825).
