# Supplementary material for: Life Satisfaction and Instagram Addiction among University Students during the COVID-19 Pandemic: The Bidirectional Mediating Role of Loneliness
Source: Int J Environ Res Public Health. 2022 Jul 9;19(14):8414. doi: 10.3390/ijerph19148414 (PMC9316946; doi:10.3390/ijerph19148414)
Supplement: Supplementary file 1 [file ijerph-19-08414-s001.zip › ijerph-1774230-supplementary.pdf]

SUPLEMENTARY MATERIALS

Table S1. Descriptive statistics (*N* = 954)

Variable	Range	*M*	*SD*	Median	Skewness	Kurtosis
Instagram addiction	6-30	16.43	6.41	16	0.23	–0.88
Life satisfaction	5-35	20.71	6.74	21	–0.24	–0.65
Loneliness general	0-11	5.65	3.52	6	–0.04	–1.26
Emotional loneliness	0-6	3.26	2.09	3	–0.15	–1.32
Social loneliness	0-5	2.39	1.78	2	0.10	–1.35

Table S2. Model A validity measures for Instagram addiction, life satisfaction, and general loneliness (*N* = 954)

					Correlations *r*			HTMT	
Variable	α	CR	AVE	MSV	1	2	3	1	2
1. Instagram addiction	0.865	0.860	0.509	0.030	**0.713**				
2. Life satisfaction	0.882	0.888	0.615	0.249	–0.157***	**0.784**		0.000	
3. Loneliness general	0.867	0.898	0.815	0.249	0.172***	–0.499***	**0.903**	0.154	0.000

Note. α = Cronbach's α, CR = composite reliability, AVE = average variance extracted, MSV = maximum shared variance, HTMT = heterotrait-monotrait, numbers in bold are the square root of the AVE. ****p* < 0.001

Table S3. Model B validity measures for Instagram addiction, life satisfaction, emotional loneliness, and social loneliness (*N* = 954)

					Correlations *r*				HTMT		
Variable	α	CR	AVE	MSV	1	2	3	4	1	2	3
1. Instagram addiction	0.865	0.860	0.509	0.038	**0.713**						
2. Life satisfaction	0.882	0.888	0.615	0.232	–0.157***	**0.784**			0.000		
3. Emotional loneliness	0.798	0.799	0.400	0.656	0.195***	–0.481***	**0.633**		0.196	0.000	
4. Social loneliness	0.790	0.809	0.461	0.656	0.098*	–0.415***	0.810***	**0.679**	0.090	0.000	0.828

Note. α = Cronbach's α, CR = composite reliability, AVE = average variance extracted, MSV = maximum shared variance, HTMT = heterotrait-monotrait, numbers in bold are the square root of the AVE. **p* < 0.05, ****p* < 0.001.

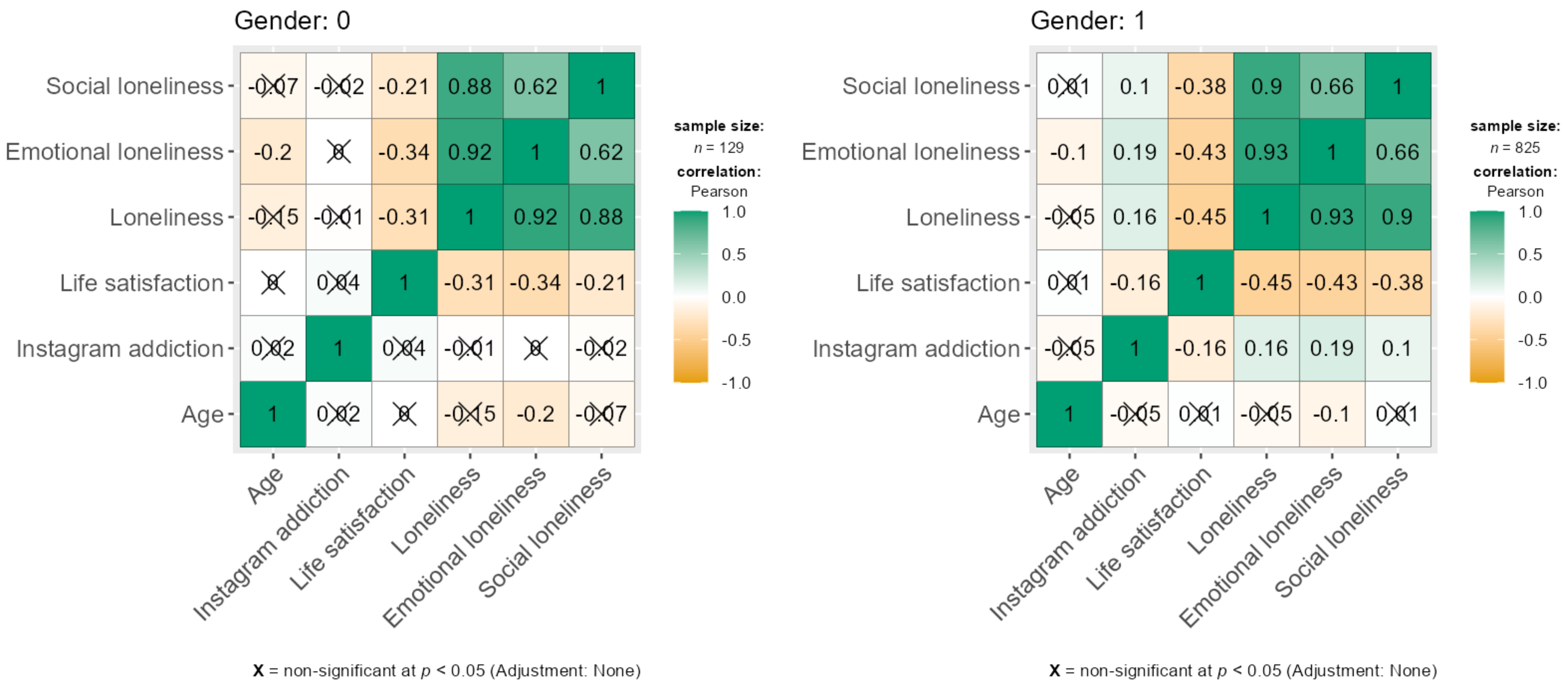

Figure S1. Correlation matrix for gender differences (Men = 0, *n* = 129; Women = 1, *n* = 825).
